# Hold on Tight! Linking Emotions and Actions in the Infant Brain

**DOI:** 10.1111/infa.70029

**Published:** 2025-07-12

**Authors:** Elisa Roberti, Chiara Turati, Ermanno Quadrelli, Stefanie Hoehl

**Affiliations:** ^1^ Psychology Department University of Milano‐Bicocca Milano Italy; ^2^ Neuromi Milan Center for Neuroscience Milano Italy; ^3^ Faculty of Psychology University of Vienna Vienna Austria

**Keywords:** actions, electroencephalography, emotions, infant cognition, social referencing

## Abstract

By the end of the first year, infants use others' emotions to interpret events, integrate social cues and build expectations on how people should behave (e.g., through *social referencing).* Yet, little is known about the neural correlates of linking others' emotions to following actions. This priming study investigates 10‐month‐old infants' electrophysiological responses to happy and disgusted emotional displays toward novel objects (prime) and subsequent actions (pushing away or pulling objects closer; target). Event‐related potentials from 30 infants showed neural responses associated with emotional processing of the prime, such as heightened attentional response (Nc) and greater cognitive processing (Pc) in response to happiness over disgust. The target action of pushing away objects elicited increased slow wave activity when following happiness. Additionally, a significant mu‐rhythm desynchronization, indicating motor resonance, was observed for pulling objects closer when preceded by happiness. Theta activity was higher for pushing away objects, indexing this as an unexpected event. These findings indicate that by 10 months, infants attend to emotional cues and use these cues to form predictions about subsequent actions. These neural correlates of bridging emotions and actions before 12 months of life reveal early neural sensitivity for processing social cues in complex contexts.

## 1 Introduction

As human beings, understanding actions is fundamental for predicting events, modulating everyday behavior and sustaining social connections. From very early on in life, we use a vast amount of information to predict action goals (Hunnius and Bekkering 2014; Von Hofsten et al. 2007) and the unfolding of social interactions (Adamson and Frick 2003; Sacheli et al. 2023). In particular, other than indicating what another person is feeling and thinking, emotional displays can be a resource for forming such predictions in specific social situations. This is already evident in the first year of life: by 6 months of age, infants attribute greater saliency to actions performed in an emotional context rather than a neutral one (Addabbo et al. 2021; Addabbo and Turati 2020). The early sensitivity to emotional cues paves the way for a more sophisticated use of emotional information later in development. For instance, by 12 months of age, infants actively use emotional information provided by adults they perceive as most knowledgeable, particularly facial expressions and vocalizations, to model their behavior in novel situations (Moses et al. 2001; Stenberg 2009). This phenomenon, known as *social referencing,* serves as a critical mechanism for integrating emotional information with motor skill development, enabling infants to learn from the expertise of their caregivers and safely explore their environment. For example, infants cross more often a deceptive drop (with a Plexiglas surface providing invisible support) if their mother expresses happiness or interest instead of fear or anger (Sorce et al. 1985; Vaish and Striano 2004).

A number of studies also described how infants build inferences about objects relying on others' social signals (Hoehl et al. 2008), and emotional expressions (Gergely et al. 2007; Hoehl and Striano 2010). For instance, 18‐month‐old infants are more inclined to approach an object when adults express pleasure rather than disgust (Moses et al. 2001). Phillips et al. (2002) found that when actors display a positive emotion toward an object, 12‐month‐old infants expect them to reach for the same object rather than a new one (Phillips et al. 2002). Barna and Legerstee (2005) extended these findings to negative emotions, showing that when a person looks at an object while being unhappy, 9‐ to 12‐month‐old infants do not expect them to pick it up (Barna and Legerstee 2005). Similarly, 10‐ to 13‐month‐old infants use their parents' fearful expressions to inhibit their approach to a toy (Walden and Ogan 1988), and 10‐month‐old infants expect a person to display a facial configuration associated with anger rather than happiness after a person takes away a desired toy (Ruba et al. 2020). One study looked at pupil dilation (indexing increased levels of sympathetic arousal related to cognitively demanding tasks) while presenting actors displaying happiness or anger either patting (positive action) or hitting (adverse action) a toy in 10‐ and 14‐month‐old infants. While both groups showed increased pupil dilation when an angry actor performed a positive action, only the older infants showed a similar response when a happy actor performed the incongruent action. These results suggest that by the beginning of the second year of life, infants associate happiness with expectations of playful behavior (Hepach and Westermann 2013).

Thus, behavioral evidence suggests that at least by 10 months of age infants use emotional contexts to form expectations about others' actions. However, less is known about the neural correlates underlying these abilities. Only one electrophysiological study (Carver and Vaccaro 2007) investigated how infants use emotional expressions to guide their behavior in novel situations. In that study, 12‐month‐old infants were exposed to emotional signals provided by the caregiver or an experimenter each time they referenced a novel object. Infants’ neural activity revealed a greater allocation of attention to novel toys associated with a disgust display than to those associated with a positive emotion or a neutral display. Yet, the phase in which infants were exposed to the emotion‐action association and the phase in which the recording of evoked potentials was conducted were about 20 min apart. Additionally, ERPs were analyzed in response to pictures of the toys seen during the behavioral testing rather than toward the action following the emotion‐action binding.

As a result, no study has directly examined the neural mechanisms underlying the immediate or near‐immediate connection between observing others' emotional reactions toward a novel object and subsequent actions in infants. This leaves a gap in our understanding of how the brain processes emotional cues in real time and how they directly influence subsequent expectations of others’ behavior.

With the present study, we aim to fill this gap by looking at 10‐month‐old infants' electrophysiological (EEG) brain activity in response to displays of happy and disgusted emotions toward an unfamiliar object and the observation of a subsequent approaching or withdrawing action to the same object. Above and beyond behavioral measures used in previous research, neuroscientific measures enable us to explore the role of different cognitive processes, including attention orienting processes driven by emotional salience (updating of) action predictions, and motor resonance (see below). We used a priming paradigm (Ishikawa et al. 2021; Nava and Turati 2020), with the purpose of understanding how emotional expressions toward an object influence the processing of actions presented afterward. It must be pointed out that the priming paradigm used here did not examine the effect of a very rapid or even subliminal presentation on attention to a later stimulus, but rather the ability to learn an association between the information presented a short distance apart. A similar approach has been already employed with infants (Otte et al. 2015; Rajhans et al. 2016). Happiness and disgust displays were chosen relying on previous studies both on social referencing (Carver and Vaccaro 2007) and the use of others’ emotions as broad attentional cues to predict their actions (Vaish and Woodward 2010). The choice of disgust over anger was due to the fact that anger could elicit both approach and withdrawal behaviors (Harmon‐Jones et al. 2013), while disgust is exclusively linked to withdrawal/avoidance motivation (Ruba et al. 2017).

First, we aimed at confirming that happiness and disgust are processed differently in infants' brains (**aim 1**). Potential changes in the amplitudes of the following event‐related potentials (ERPs) could be expected. At earlier latencies (faster and more automatic processes), a Negative Central (**Nc**), a component that when enhanced reflects greater attentional processing, was previously described at frontal and central electrode sites in response to emotional displays (Grossmann et al. 2006; Quadrelli, Conte, et al. 2019). Another component, the Positive central (Pc) that follows the Nc (i.e., in the 600–1000 ms time window) has been described in central and parietal electrodes with a larger amplitude in response to familiar items (Grossmann et al. 2006).

We then hypothesized that 10‐month‐old infants are using emotional displays to make predictions about others' actions based on their emotions (**aim 2**). In this case, we expected to find different neural activations for congruent and incongruent emotion‐action displays. This activation could be similar for the two emotions in case they both function as a broader attentional cue, or different for happiness and for disgust in case they serve as a semantic context to interpret subsequent actions (Addabbo and Turati 2020; Vaish and Woodward 2010). A larger Nc for incongruent prime‐target pairings was previously described in priming paradigms (Peykarjou et al. 2020) and in response to unexpected actions (Langeloh et al. 2020) in infants. In similar paradigms with adult populations, a late slow wave (LSW) was found as well at slightly later latencies, reflecting increased attention toward unexpected targets, or novelty detection (Chennu et al. 2013). This LSW has been observed in infants as well (around 900 ms post‐stimulus onset, at frontal, frontocentral, and parietal sites), with lower amplitudes in response to novel stimuli (Richards 2003; Wiebe et al. 2006).

In addition to ERPs, brain oscillations are a powerful tool for investigating socio‐cognitive processes. Specifically, the alpha oscillatory band (6–9 Hz in infants) (Debnath et al. 2019) over somatosensory cortex (i.e., central electrodes) can index the prediction of a motor response. A differential alpha suppression (also called mu‐rhythm desynchronization) for the two presented actions would support the hypothesis that these are encoded as substantially different motor plans (Addabbo et al. 2020; Köster, Langeloh, Kliesch, et al. 2020; Quadrelli, Roberti, et al. 2019; Southgate et al. 2009), even more so when embedded in a social context (Meyer et al. 2022).

In parallel, a few studies described a heightened activation in the theta rhythm in response to unexpected events (Köster et al. 2019; Köster et al. 2021), learning processes (Begus and Bonawitz 2020) and social processing (Van Der Velde et al. 2021). This rhythm is typically explored in the whole time‐windows and for all regions of interest (Köster et al. 2021). We adopted a similar exploratory approach with the hypothesis that a different theta response for the two actions could be expected, either depending on the emotional context or on the familiarity of the action. Details of this analysis and results are presented in the Supporting Information S1.

The study was preregistered (https://aspredicted.org/JC9_L87). Notably, although the main hypotheses and study design are the same, a few of the components reported here differ from the ones mentioned originally. After preregistration, further literature review and data analysis lead to reconsidering a few aspects. Specifically, the analysis of the ERPs in the priming phase was added at a later date to ensure that the two emotions were indeed differentiated. Moreover, an N400 component was initially hypothesized as a response to the incongruent trials in the target phase (such a component had previously been reported as related to mismatch of multimodal information in older children, Ke et al. 2022) or a late positive component (Pc) indicating an updating working memory process (Grossmann et al. 2006). Yet, the visual inspection of the ERP waveforms revealed a response in a wider and later (800–1100 ms) time window than typically associated with an N400 or a classical Pc. This protracted, late‐latency activity aligns more closely with phenomena reported in the literature as Late Slow Waves (LSW), often implicated in high‐level cognitive processes such as working memory updating rather than semantic integration of information. Consistent with literature describing similar late‐latency, broadly distributed activities associated with such processes (Roth and Reynolds 2022), we labeled this component as LSW.

## 2 Methods

### 2.1 Participants

Data was collected between August 2019 and February 2020. Mothers were recruited in local hospitals in the neonatal units or during mother‐infant activity classes. All families were German‐speaking and parents predominantly reported high education levels (more than 90% held a university degree). All infants were born full term (between 37 and 42 gestational weeks), with a normal birth weight (> 2500 g) had intact vision and hearing abilities and no history of neurological or significant medical conditions. Thirty 10‐month‐old infants (*M* = 10 months 15 days, SD = 14 days, 10 girls and 20 boys) were included in the analyses, as determined by an a priori power analysis a repeated‐measures ANOVA with medium effect size of *f* = 0.25, power of 0.80 and correlation among repeated measures around 0.6–0.7. Additional 20 infants were tested but excluded from the final sample due to fussiness (*n* = 9), artifacts resulting in an insufficient number of analyzable trials (*n* = 9), or technical problems (*n* = 2). This attrition rate of around 40% is in line with other EEG infant studies (DeBoer et al. 2013; Stets et al. 2012), especially those that used similar paradigms, highly demanding for infants' attention (Righi et al. 2014). The ethical committee of the University of Vienna approved the study (Approval n. 00,427, June 11, 2019), and parents gave their informed written consent before starting the experiment, according to the ethical standards of the Declaration of Helsinki (“World Medical Association Declaration of Helsinki” 2013). Parents received 6 euros as reimbursement for their travel expenses, and infants received a small toy and a certificate of participation.

### 2.2 Design and Stimuli

**FIGURE 1:**
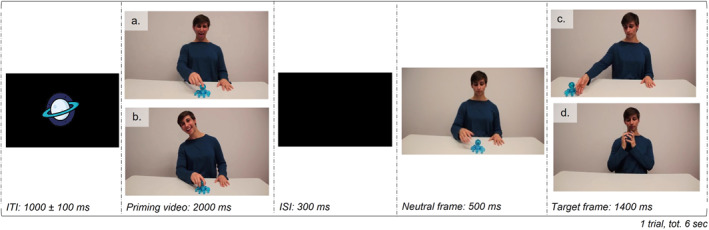
Trial structure and duration of inter‐trial intervals (ITI), priming videos (a. *disgust*; b. *happiness*; in both cases a reach only action was performed), inter‐stimuli intervals (ISI), neutral frame, and target frames (c. *push*; d. *pull*). One priming video and one target frame per trial were presented; two examples are given to indicate the stimuli presented under the different conditions. The actors' expression was neutral during both the neutral and the target frames, while their gaze in the priming videos was directed at the observer at the beginning of the video, then shifted to the object at the start of the movement and returned to the observer when the emotion was expressed.

The priming paradigm consisted of a 2000 ms priming video (i.e., happiness or disgust display toward an object) followed by a 300 ms inter‐stimuli interval, a 500 ms neutral frame, and a 1400 ms target frame (i.e., the same actor either holding or pushing away the same object) (Figure 1). A random inter‐trial interval between 900 and 1100 ms was set between two trials.

#### 2.2.1 Priming Videos

To convey information about an emotional display toward an object, we recorded 2000 ms videos of two actors (one male, one female) expressing happiness or disgust toward six different unfamiliar objects. The objects chosen were a blue plastic octopus, an orange funnel, a green sponge, a red pepper mill, an orange plastic hedgehog, and a purple toy with a handle. These objects were chosen, as they are not too familiar for infants, in order to avoid a pre‐existing affective valence. The images of the chosen objects are available online at the following link: https://doi.org/10.17605/OSF.IO/GEFST. At the beginning of the scene, all the objects were placed at the center of the screen while the actors made eye contact with the observer to ensure attention engagement. After five frames, the actors looked at the object and started approaching it with their hand, reaching it at around 734 ms (22 frames). As the contact happened, the actor expressed one of two emotions, happiness and disgust, through the face, voice, and body, while their gaze shifted back to the observer.

#### 2.2.2 Target Frames

After a neutral frame of the actor looking with a neutral expression at the same object shown in the priming phase was presented for 500 ms, the target frame appeared. One of two scenarios could be presented: either the actor (again, with a neutral expression) would be holding the object closer to the body or pushing it away. The side where the object was moved was counterbalanced across trials. The target frames were either congruent or incongruent with the priming stimuli, resulting in four conditions: *happiness‐pull* (congruent), *happiness‐push* (incongruent), *disgust‐pull* (incongruent), *disgust‐push* (congruent).

### 2.3 Procedure

Testing was performed in a dimly lit room and to avoid possible distractions, participants were surrounded by blue curtains, hiding everything except from the screen to their sight. The stimuli were presented on a BENQ 21.5″ screen (1920 × 1080‐pixel resolution, 60 Hz refresh rate). Infants sat approximately 60 cm from the screen in their caregiver's lap, forming a maximum visual angle of 19.51° vertically and 18.27° horizontally. The stimuli were presented in a pseudo‐random order using an Opensesame script (https://osdoc.cogsci.nl/) so that no more than three consecutive equivalent emotions and no more than three congruent or incongruent consecutive pairs were presented. The total number of trials was 96, for a total duration of around 10 min. A break could be taken when needed, and the procedure was stopped when infants showed signs of distress or whenever parents asked to interrupt. The mean number of trials presented was 92.87 (SD = 8.07) (*happiness‐pull*: *M* = 23, SD = 2.6; *happiness‐push*: *M* = 23.3, SD = 2.1; *disgust‐pull*: *M* = 23.5, SD = 1.4; *disgust‐push*: *M* = 23.2, SD = 2). As shown by a non‐parametric repeated‐measures ANOVA (Friedman test), the number of presented trials did not differ between the four conditions (*χ* ^*2*^ (3) = 6.17, *p* = 0.1). Parents were asked not to interact with their infants to minimize the influence of external social cues.

### 2.4 Electroencephalogram Recording and Analyses

EEG was recorded continuously using a 32‐channel ActiCap System (Brain Products GmbH, Gilching, Germany), with active electrodes arranged according to the 10‐20 system, amplified through a BrainAmp amplifier, and recorded with BrainVision Recorder and Video Recorder software (Brain Products GmbH, Gilching, Germany). Impedances of the electrodes were checked before the beginning of the recording and were considered acceptable if lower than 20 kΩ. The signal was referenced online to the right mastoid (REF) reference channel, and data was sampled at 500 Hz. Further offline processing was performed using Matlab, EEGLAB toolbox (Delorme and Makeig 2004). The signal was band‐pass filtered (0.3–30 Hz) and re‐referenced to the average of the right and left mastoids.

Different segmentation and preprocessing steps followed according to each of the planned analyses. For the ERPs analyses, data were segmented with a 100 ms baseline before (Patzwald et al. 2020) and 2000 ms after the onset of the videos and then the signal was corrected with respect to the average voltage of the baseline. All the trials where the signal exceeded a voltage threshold of 200 μV within a 200 ms interval in the channels of interest (Michel et al. 2019) were marked as bad. Artifacts, such as blinks, eye movements or other movements which cannot be automatically individuated, were again manually checked and, if necessary, rejected. A maximum of three individual bad channels of interest were replaced using spherical spline interpolation. Only the trials in which the prime was watched for more than 50% were included in the final analysis, to maximize the possibility that the emotional information was processed.

Across participants, an average of *M* = 11.1 trials per condition (SD = 2.6, range 6–20) contributed to the average ERPs in response to **priming videos** (*happiness*: *M* = 10.2, SD = 2.9, range 6–20; *disgust*: *M* = 10.4, SD = 3.1, range 6–20). Due to the longer time window, *n* = 4 subjects presented data with too much noise and had to be excluded from further analyses because they presented a low number of trials per condition (*happiness*: *M* = 5.2, SD = 1.8; *disgust*: *M* = 5, SD = 2). The final sample included in the ERP analyses was therefore of 24 participants. An average of *M* = 11.1 trials per condition (SD = 2.6, range 8–19) contributed to the average ERPs in response to the **target frames** (*happiness*‐*pull*: *M =* 11.3, SD = 2.6, range 8–19; *happiness‐push*: *M* = 10.8, SD = 2.7, range 8–19; *disgust‐push*: *M* = 10.3, SD = 2.4, range 8–19; *disgust‐pull*: *M* = 11.8, SD = 2.6, range 8–19). The number of rejected trials is mainly due to the length of the trials. Especially in the second half of the procedure when infants' attention naturally diminishes, eye and body movement happened more frequently during the target stimuli presentation. The number of trials that contribute to the final analyses is nonetheless in line with previous research using similar paradigms (Crespo‐Llado et al. 2018; Hendrickson et al. 2019). Individual averages were then computed separately for each channel across all trials within each condition.

For the **time‐frequency** analyses, data were segmented into trials from 1000 ms before the onset of the target and 1800 ms following the target stimulus onset. Trial and artifact rejection, as well as bad channel interpolation and inclusion of trials only watched for more than 50% were performed as described for the ERP analyses. Due to the longer time window, *n* = 6 subjects presented data with too much noise and had to be excluded from further analyses because they presented a low number of trials per condition (*happiness‐pull*: *M* = 2, SD = 2.35; *happiness‐push*: *M* = 4, SD = 2; *disgust‐pull*: *M* = 3.94, SD = 1.87; *disgust‐push*: *M* = 2.8, SD = 2.59). The final sample of included participants was therefore of 24 subjects (6 girls and 18 boys). Across participants, an average of *M* = 11.6 trials per condition (SD = 0.29, range 7–22) contributed to the grand average (*happiness‐pull*: *M* = 11.63, SD = 3.03, range 7–22; *happiness‐push*: *M* = 10.88, SD = 2.76, range 7–17; *disgust‐push*: *M =* 11.88, SD = 2, range 7–21; *disgust‐pull*: *M =* 11.88, SD = 3.44, range 7–22). Time‐frequency analyses were performed on each artifact‐free trial using continuous wavelet transform with Morlet wavelets at 1 Hz intervals in the 3–20 Hz range. The first and last 400 ms of each segment were removed to eliminate distortion created by the wavelet transform. A 500 ms baseline period from 600 to 100 ms before stimulus onset was selected. This baseline occurred during the neutral frame presented, which contained similar and equally interesting information to the experimental stimuli, but not conveying the variable of interest (De Klerk and Kampis 2021). The average activity in the 6–9 Hz (Langeloh et al. 2018) range during the 500 ms baseline was subtracted from average activity recorded during the target presentation. Average wavelet coefficients within infants were calculated using the mean across the trials.

The specific time windows and clusters analyzed for each of the three analyses are detailed in the following paragraphs.

#### 2.4.1 ERPs—Priming Videos

Although this was not initially planned when the study was preregistered, an analysis of the ERPs evoked by the priming videos was first performed, to ensure that the two displays were indeed processed differently in infants' brains (**aim 1**).

Through a visual inspection, a first deflection was observed between 200 and 400 ms across all locations, followed by a return to baseline. This deflection happened before the onset of the emotion (22^nd^ frame, 734 ms). One sample *t*‐tests showed that this negative deflection was significantly different than the baseline at frontal (*t* (23) = −3.40, *p* = 0.002, *d* = −0.7), frontocentral (*t* (23) = −3.44, *p* = 0.002, *d* = −0.7), central (*t* (23) = −3.51, *p* = 0.002, *d* = −0.72) and parietal (*t* (23) = −2.78, *p* = 0.011, *d* = −0.57) ROI. No differences between the two conditions were found (all *p* > 0.4). Hence, in the results section, results are presented referring to the onset of the emotional display (indicated as 0 ms), up to the end of the video (indicated as 1266 ms).

The target ERPs in response to the emotional displays were the Nc and a later Pc. For the Nc, typically analyzed clusters are frontal (F3, F4), frontocentral (FC3, FC4), and central (C3, C4), while the Pc is usually found at central (C3, C4) and parietal (P3, P4) electrodes (Grossmann et al. 2006; Jessen and Grossmann 2017). Midline electrodes were not included in the analysis as there is large literature discussing the possible hemisphere specialization when processing emotional stimuli (Huang et al. 2021; Pane et al. 2019; Rotem‐Kohavi et al. 2017). Given the interest in detecting possibly lateralized effects, the inclusion of midline electrodes could confound significant hemispheric differences.

#### 2.4.2 ERPs—Target Frames

The emotion—action link processing was then explored by analyzing both ERPs time‐locked to the target frames and the mu‐rhythm desynchronization and theta band activity in the time‐frequency domain (**aim 2**).

The clusters of electrodes corresponding to each region of interest (ROI) were as follows: frontal (F3, F4); frontocentral (FC3, FC4), central (C3, C4), and parietal (P3, P4). The Nc is typically observed over frontal and central sites, while the LSW is generally recorded at central and parietal ROI, sometimes extending in frontocentral areas (Geangu et al. 2015; Grossmann et al. 2005, 2005, 2006). Note that in this case, similarly to the priming analyses, midline was not included for the same reason, deviating from the pre‐registration. The frontocentral channels (FC3, FC4) were not mentioned in the pre‐registration due to oversight, but we decided to include them in the analysis given their spatial proximity to the pre‐registered channels and the fact that the Nc is often reported on these channels (e.g., Hoehl et al. 2008).

#### 2.4.3 Mu‐Rhythm Analysis—Target Frames

To further explore the link between emotional displays and others' actions (**aim 2**), the neural response to the target frames in terms of time‐frequency activation was explored.

Like in previous studies, a mu‐rhythm desynchronization (6–9 Hz) in response to the actions in the target frames over central electrodes (C3, Cz, C4) could be expected (Langeloh et al. 2018; Marshall and Meltzoff 2011; Quadrelli et al. 2021; Quadrelli et al. 2019). We predicted an early desynchronization, in the 100–400 ms time window, in line with previous studies suggesting an early activation of motor areas in response to action perception (Lepage et al. 2010; Liao et al. 2015; Quadrelli et al. 2019). Moreover, early activation can also be expected since infants saw several repetitions of the trials, and each target frame was preceded by a neutral frame creating an anticipation of the action. Additionally, previous literature has shown activity in the alpha band in occipital clusters as related to visual attention, not differing between conditions (Debnath et al. 2019; Warreyn et al. 2013). Therefore, we also analyzed the channels over the occipital region (O1, Oz, O2). The midline was included here as it was previously found to be involved in action processing in the frequency domain (Van Elk et al. 2008).

### 2.5 Statistical Analysis

Planned statistical analyses aimed at comparing ERPs amplitude, theta and mu‐rhythm in the different conditions through repeated‐measures analyses of variance (ANOVAs) performed in Jamovi (The jamovi project 2019, https://www.jamovi.org) (Version 2.3.28).

Post‐hoc comparisons were performed for all significant main effects and interactions and corrected for multiple comparisons (Bonferroni *p* values are presented). All statistical tests were interpreted at a 0.05 level of significance (two‐tailed). The Greenhouse‐Geisser correction was applied whenever the assumption of Sphericity was violated (indicated by *ε*). All dependent variables were checked and found to be normally distributed (Shapiro‐Wilk *p* > 0.05).

## 3 Results

### 3.1 ERPs—Priming Videos

**FIGURE 2:**
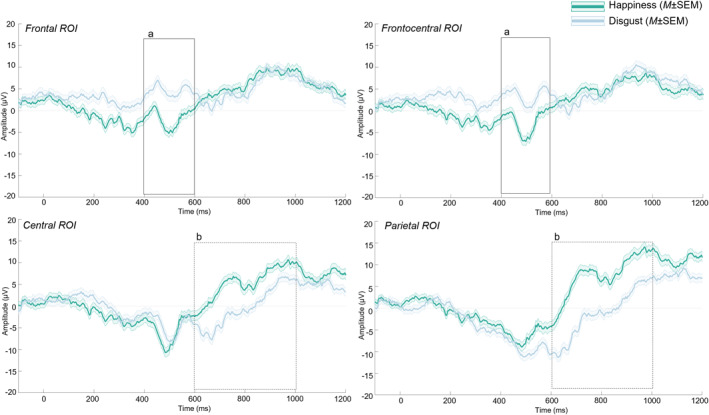
ERPs in response to the emotional displays presented in the priming videos. (a) Negative deflection (Nc) in the 400–600‐ms latency interval in the frontal (left panel) and frontocentral (right panel) electrodes; (b) positive waveform (Pc) in the 600–1000 ms latency interval at central (left panel) and parietal (right panel) electrodes.

From the grand average of all participants, a negative deflection (**Nc**) in the **400–600‐ms** latency interval in the frontal and frontocentral ROI and a positive waveform (**Pc**) in the **600–1000 ms** latency interval at central and parietal ROI were confirmed in response to the priming videos (Figure 2).

**FIGURE 3:**
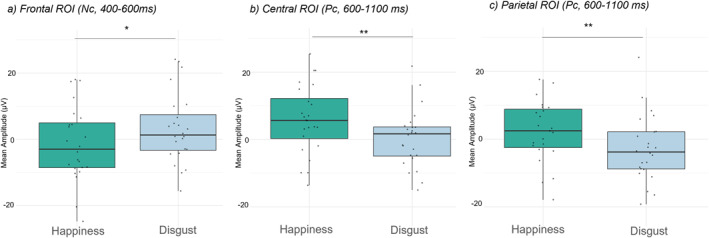
Mean amplitudes, for (a) frontal (b) central and (c) parietal electrodes for the two conditions (*happiness* and *disgust*). ***p < 0.001; **p < 0.01; *p < 0.05.

For the Nc, an omnibus ANOVA was first performed with ROI (frontal, frontocentral, and central electrodes), emotion (*happiness*, *disgust*), and lateralization (left and right electrodes) as within‐subject factors. A significant main effect of ROI was found, *F* (2,46) = 17.82, *p* < 0.001, *η* ^2^_p_ = 0.44. The post‐hoc comparison showed that while the frontal and frontocentral activity did not differ, the activation over the central region was lower (*M* = −4.03 μV; SD = 8.97) than for the other regions (frontal: *M* = 0.84 μV; SD = 9, *t* (23) = −4.43, *p* > 0.001, *d* = −0.92, frontocentral: *M* = 0.15 μV; SD = 9.45, *t* (23) = −5.42, *p* > 0.001, *d* = −1.13). This main effect was further qualified by an interaction between emotion and ROI *F* (2,46) = 4.54, *p* = 0.016, *η* ^2^_p_ = 0.17. In particular, *disgust* had a lower activation in central electrodes (*M* = −4.02 μV; SD = 8.78) if compared with frontal (*M* = 0.84 μV; SD = 9.26 (*t* (23) = −5.66, *p* > 0.001, *d* = −1.18) and frontocentral (*M* = 0.15 μV; SD = 8.87, *t* (23) = −5.87, *p* > 0.001, *d* = −1.22). All other main effects and interactions were non‐significant (*p* > 0.1). Given the variability of voltage within the same areas, separate repeated‐measures ANOVAs were performed within each region of interest, with emotion (*happiness, disgust*), and lateralization (left, right electrodes) as factors. In the **frontocentral and central ROI** no main effects or interactions were found (all *p* > 0.1). In the **frontal ROI** a significant main effect of emotion (*F* (1,23) = 4.35, *p* = 0.048, *η* ^2^_p_ = 0.16) was found, with *happiness* evoking a greater negative response (*M* = −1.50 μV; SD = 11.30) than *disgust* (*M* = 3.17 μV; SD = 10.60) (*t* (23) = −2.09, *p* = 0.048, *d* = −0.43) (Figure 3a). No other main effect or interactions were found (all *p* > 0.40). Therefore, this component indicates a heightened infant attention to *happiness* displays.

For the Pc, an omnibus ANOVA was first performed with ROI (central and parietal electrodes), emotion (*happiness*, *disgust*), and lateralization (left and right electrodes) as within‐subject factors. A significant main effect of emotion was found, *F* (1,23) = 12.24, *p* = 0.002, *η* ^2^_p_ = 0.35. The post‐hoc comparison showed that the average voltage for *happiness* (*M* = 4.78 μV; SD = 10.5) was higher than for *disgust* (*M* = −1.25 μV; SD = 8.8, *t* (23) = 3.50, *p* = 0.002, *d* = 0.71). A significant main effect of ROI was also found, *F* (1,23) = 7.18, *p* = 0.013, *η* ^2^_p_ = 0.24. The post‐hoc comparison showed that while the frontal and frontocentral activity did not differ, the activation over the parietal ROI was lower (*M* = 0.45 μV; SD = 9.96) than for the central region (*M* = 3.08 μV; SD = 8.48, *t* (23) = −2.68, *p* = 0.013, *d* = −0.55). All other main effects and interactions were non‐significant (*p* > 0.1).

Again, given the high variability within the same area, separate ANOVAs were then performed for the two ROIs with emotion (*happiness, disgust*), and lateralization (left, right electrodes) as factors. At **central ROI,** a main effect of emotion was found (*F* (1,23) = 10.1, *p* = 0.004, *η* ^2^_p_ = 0.31). Post‐hoc comparisons showed that the average voltage for *happiness* (*M* = 5.82 μV; SD = 10.200) was higher than for *disgust* (*M* = 0.33 μV; SD = 8.7) (*t* (23) = 3.18, *p* = 0.004, *d* = 0.65). No other main effect or interactions were found (all *p* > 0.40) (Figure 3b). Similarly, at **parietal ROI,** results revealed a main effect of emotion (*F* (1,23) = 10.77, *p* = 0.003, *η* ^2^_p_ = 0.32). Post‐hoc comparisons showed that the average voltage for *happiness* (*M* = 3.73 μV; SD = 11.600) was higher than for disgust (*M* = −2.84 μV; SD = 9.78) (*t* (23) = 3.28, *p* = 0.003, *d* = 0.67) (Figure 3c). No other main effect or interactions were found (all *p* > 0.80). In this case, the Pc reflects greater higher‐level cognitive processing for happiness.

### 3.2 ERPs—Target Frames

**FIGURE 4:**
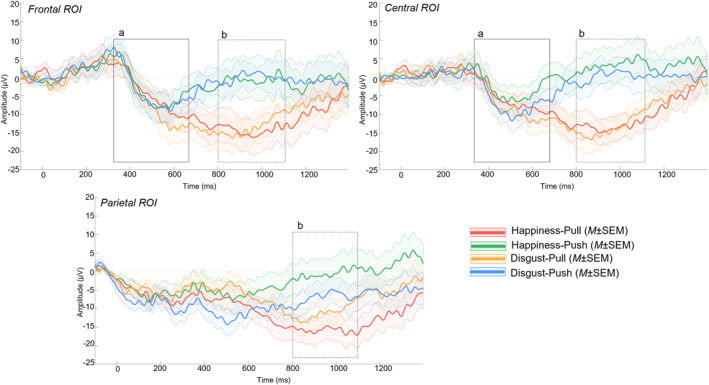
ERPs in response to the action displays (presented separately according to the preceding emotional display) presented in the target frames. (a) Negative deflection (Nc) in the 350–650 ms latency interval in the frontal (upper left panel) and central (upper right panel) electrodes; (b) late slow wave (LSW) in the 800–1100 ms in all ROI.

As predicted when planning the study, from the grand average of all participants, a negative deflection (**Nc**) in the **350–650 ms** latency interval in the frontal and central ROI was detected. The initially predicted N400 and Pc were not detected in the ERP waveforms and were therefore not analyzed. Moreover, a late slow wave (**LSW**) in the **800–1100 ms** interval in all ROI was observed in response to the target frames depicting holding or pushing away actions (Figure 4). Analysis of this component was not initially planned and should therefore be considered exploratory.

For the Nc (350–650 ms window), in parallel with what was done for the priming analysis, an omnibus ANOVA was first performed with ROI (frontal, frontocentral, and central electrodes), emotion (*happiness*, *disgust*), action (*pull, push*) and lateralization (left and right electrodes) as within‐subject factors. This analysis did not yield significant results. However, Mauchly's Test of Sphericity was statistically significant for ROI (*W* = 0.55, *p* < 0.001), indicating that the variances of the different ROIs are not equal. This indicates that pooling the data might not be optimal to detect differences at the other factors' levels. Therefore, repeated‐measures ANOVAs were performed for each region of interest separately, with emotion (*happiness*, *disgust*), action (*pull*, *push*), and lateralization (left electrodes, right electrodes) as within‐subject factors. These ANOVAs confirmed the absence of significant main effects or interactions at **frontal** (all *p* > 0.37), **frontocentral** (all *p* > 0.12), and **central** ROI (all *ps* > 0.11), indexing a similar attentional response to all conditions.

For the LSW (800–1100 ms time window), the omnibus ANOVA with ROI (frontal, frontocentral, central and parietal electrodes), emotion (*happiness*, *disgust*), action (*pull, push*) and lateralization (left and right electrodes) as factors yielded a significant main effect of action, *F* (1,29) = 19.39, *p* < 0.001, *η* ^2^_p_ = 0.4. This main effect was further qualified by an interaction between action and ROI (*F* (3,87) = 3.80, *p* = 0.013, *η* ^2^_p_ = 0.12), as well as between emotion, action and ROI (*F* (3,87) = 5.21, *p* = 0.002, *η* ^2^_p_ = 0.12). Given the significant three‐way interaction between action, emotion, and ROI, interpreting this effect within the omnibus model would require numerous post‐hoc comparisons, increasing the risk of Type I error. To provide a more targeted analysis, separate ANOVAs were conducted for each region to examine how emotions and actions interact.

**FIGURE 5:**
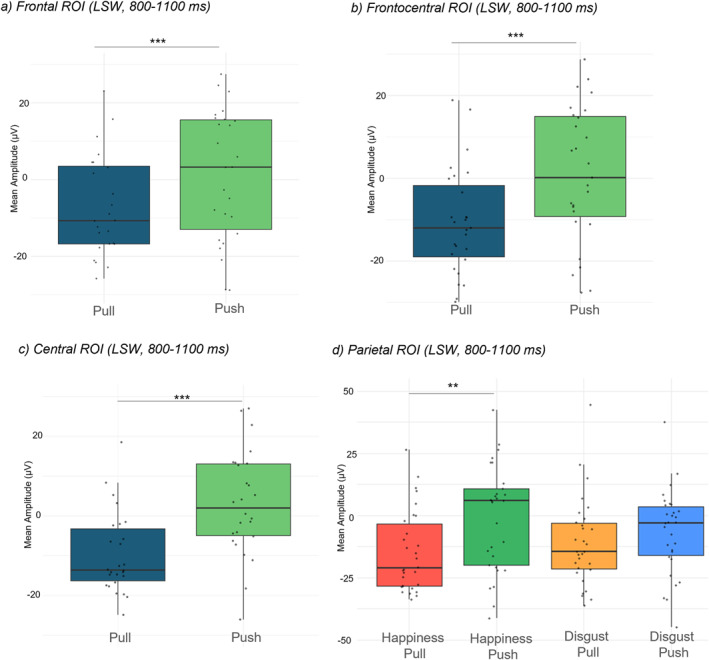
Mean amplitudes for the *pull* and *push* actions at (a) frontal, (b) frontocentral and (c) central electrodes. (d) Mean amplitudes at parietal electrodes for the four conditions. ***p < 0.001; **p < 0.01; *p < 0.05.

At the **frontal ROI,** a main effect of action was found (*F* (1,29) = 20.64, *p* < 0.001, *η* ^2^_p_ = 0.42). Pairwise comparisons showed that when infants observed the *pull* action, the mean voltage was lower (*M* = −14.10 μV; SD = 18.90) than when they observed the *push* action (*M* = −0.85 μV; SD = 21.8) (*t* (29) = −4.54, *p* < 0.001, *d* = −0.83), thus reflecting enhanced cognitive processing for the latter type of action (Figure 5a). No further main effect or interactions were found (all *ps* > 0.50). Similarly, at the **frontocentral ROI,** a main effect of action (*F* (1,29) = 18.16, *p* < 0.001, *η* ^2^_p_ = 0.38) indicated a lower voltage for the *pull* action (*M* = −14.17 μV; SD = 16.39) than for the *push* action (*M* = −0.06 μV; SD = 20.88) (*t* (29) = −4.26, *p* < 0.001, *d* = −0.78) (Figure 5b). No further main effect or interactions were found (all *ps* > 0.40). At the **central** ROI, a similar main effect of action was again found (*F* (1,29) = 22.20, *p* < 0.001, *η* ^2^_p_ = 0.43). Pairwise comparisons showed that when the action observed was *pull* (*M* = −13.41 μV; SD = 13.75), the mean voltage was lower than when the action was *push* (*M* = 2.82 μV; SD = 19.78) (*t* (29) = −4.71, *p* < 0.001, *d* = −0.86) (Figure 5c). No further main effects or interactions were found (all *p*s > 0.30).

The main effect of action was still significant at the **parietal** ROI (*F* (1,29) = 8.58, *p* = 0.007, *η* ^2^_p_ = 0.21), where again the mean voltage was lower for the action *pull* (*M* = −12.94 μV; SD = 14.3) than for the action *push* (*M* = −3 μV; *S* = 18.50) (*t* (29) = −2.93, *p* = 0.007, *d* = −0.54) (Figure 5d). Further, a significant interaction between emotion and action (*F* (1,29) = 5.76, *p* = 0.023, *η* ^2^_p_ = 0.17) indicated that, when the *happiness* display preceded the target action, the *push* action evoked higher voltage (*M* = 0.69 μV; SD = 23.40) than the action *pull* (*M* = −14.82 μV; SD = 16.40), *t* (29) = 4.05, *p* = 0.002, *d* = 0.74. Not only is the increased processing for the push action confirmed, but this result also indicates that the effect is particularly driven by those trials where an incongruent (*happiness*) prime was presented. No further main effects or interactions were found (all *p*s > 0.08).

### 3.3 Mu‐Rhythm: Target Analysis

For 24 infants, data was available for time‐frequency analysis. As planned as secondary analyses in the preregistration, the alpha (6–9 Hz) (Langeloh et al. 2018) band was analyzed. An exploratory analysis of the theta band (3–5 Hz) (Reid et al. 2009) is also available in the Supporting Information S1.

**FIGURE 6:**
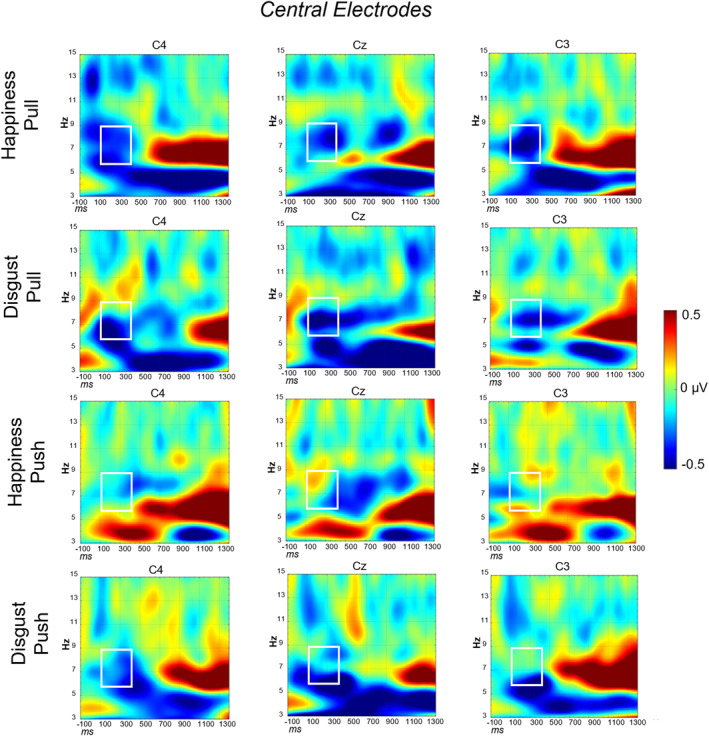
Time‐frequency plots for the four conditions for the central electrodes (left: C3, middle: Cz, right: C4). The white rectangles show the selected time window (100–400 ms) for the alpha band analyzed (6–9 Hz).

**FIGURE 7:**
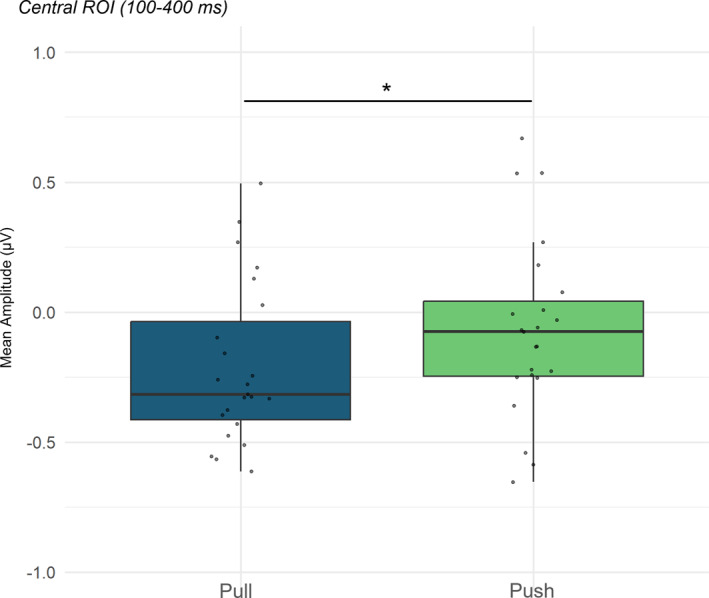
Mean amplitude at the central electrodes for the two conditions (*pull* and *push*) in the alpha band. ***p < 0.001; **p < 0.01; *p < 0.05.

A desynchronization in the 6–9 Hz band within the 100–400 ms time window was detected in the central ROI (Figure 6). The omnibus ANOVA with ROI (central and occipital electrodes), emotion (*happiness*, *disgust*), action (*pull*, *push*), and lateralization (left, right, and midline electrodes) as within‐subject factors, confirmed a main effect of ROI, *F* (1,23) = 24.29, *p* < 0.001, *η* ^2^_p_ = 0.51. The post‐hoc comparison showed that, in general, the activation over occipital electrodes was lower (*M* = −1.28 μV; SD = 1.26) than over central electrodes (*M* = −0.18 μV; SD = 0.38) (*t* (23) = −4.93, *p* < 0.001, *d* = −0.9. All other main effects and interactions did not reach significance (*p* > 0.07). Two separate ANOVAs were then performed to analyze the alpha band activity separately for the two ROIs. First, we ran an ANOVA on **central** electrodes, with emotion (*happiness*, *disgust*), action (*pull*, *push*), and lateralization (left, right, and midline electrodes) as within‐subject factors. A main effect of action was found, *F* (1,23) = 4.32, *p* = 0.049, $\eta_{p}^{2}$ = 0.16. The post‐hoc comparison showed that, for the action *pull*, the mean voltage was lower (*M* = −0.25 μV; SD = 0.37) than for the action *push* (*M* = −0.11 μV; SD = 0.40), indexing a greater mu‐rhythm desynchronization (*t* (23) = −2.08, *p* = 0.049, *d* = −0.42) (Figure 7). Here, no interaction between emotion and action was found (all other main effects and interactions did not reach significance, *ps* > 0.2). Yet, when compared to the baseline by means of one sample *t* tests, the only significant desynchronization was found for the *happiness*‐*pull* condition, both over left (*t* (23) = −2.83, *p* = 0.04, *d* = −0.5; *M* = −0.32 μV; SD = 0.64) and right electrodes (*t* (23) = −2.81, *p* = 0.02, *d* = −0.57; *M* = −0.33 μV; SD = 0.57). No other comparison was statistically significant (all *p*s > 0.08).

Moreover, since activity in the alpha band in occipital clusters was often found reflecting more generalized visual attention (Debnath et al. 2019; Warreyn et al. 2013), another ANOVA was performed on **occipital** electrodes with emotion (*happiness*, *disgust*), action (*pull*, *push*), and lateralization (left, right, and midline electrodes) as within‐subject factors. Only a main effect of lateralization was found, *F* (1.50,34.59) = 4.04, *p* = 0.037, $\eta_{p}^{2}$ = 0.15, *ε* = 0.75, with the electrode over the left hemisphere (*M* = −1.04 μV; SD = 1.05) recording a higher voltage than the one over the midline (*M* = −1.52 μV; SD = 1.35) *t* (46) = 2.84, *p* = 0.02, *d* = 0.60. All other main effects or interactions were not significant (all *ps* > 0.20), marking equal visual attention in the different conditions.

## 4 Discussion

The present study explored whether infants' neural processing relies on the emotional context to form predictions on subsequent observed actions. To do so, we presented *happiness* and *disgust* displays followed by pulling and pushing objects as target actions. Therefore, our first goal was to confirm that the two emotional displays are processed differently (**aim 1**). Indeed, the Nc revealed heightened attention in response to *happiness*, as reflected by a more negative amplitude in frontal areas. This result aligns with previous evidence for a higher sensitivity in infants' brains for *happiness* than *disgust* (Poncet et al. 2022). The later waveform (Pc) at central and parietal electrodes confirmed this observation: the enhanced processing at a higher cognitive level for *happiness* suggests greater sensitivity for information regarding happy displays.

Looking at the central question of this study, where we hypothesized that a link between emotions and actions could be indexed at a neural level in 10‐month‐old infants (**aim 2**), our results substantiated this idea. ERPs first showed a non‐specific Nc response at central locations for the target actions (i.e., the four conditions evoked a similar attentive response), while the later component (the LSW) showed a significantly enhanced positivity for pushing away an object compared to when pulling it closer. Crucially, at posterior locations we found that this response to the *push* away action was specifically detected when it was preceded by *happiness* (i.e., the more unexpected scenario). The fact that this differentiation was not found in the early time window but at later latencies suggests that higher‐level cognitive processing is engaged when infants observe something unusual; this pattern of results is consistent with several previous infant studies, although it has been differently interpreted as increased allocation of attention (Grossmann et al. 2005, 2006), enhanced sensory processing (Geangu et al. 2015), or as processing in response to motivationally relevant or arousing stimuli (Dunning and Hajcak 2009; Hill et al. 2021). Keeping an object in one's hands and looking at it might be perceived as more familiar for infants, as their typical behavior from 4 months of life is to explore objects with their hands and mouth (Gerber et al. 2010). Therefore, pushing the object away could require more cognitive resources when observed, especially if the same person previously showed happy emotionality, as previous knowledge does not facilitate its processing.

Altogether, these results seem to support our hypothesis: infants are making predictions about others' actions before the first year of life by drawing on information about experienced emotions, particularly happiness. No detection of incongruence between disgust and pulling the object closer was observed. This may be due to the fact that this association is more complex and emerges later, possibly between 12 and 19 months, where a clear understanding of disgust displays toward food has been observed at a behavioral level (Carver and Vaccaro 2007; Lopez and Walle 2022; Walle and Campos 2014). Another potential explanation is that, given the higher attentional response for happiness displays, only those might act as a cue for a relevant situation (Reschke et al. 2017). Future studies should focus on this aspect, either following up on the trajectory of disgust as a social referencing tool or investigating whether other emotions can guide attention similarly to happiness.

The difference in mu‐rhythm desynchronization goes in the same direction: a larger sensorimotor activation over central electrodes is observed in response to the more familiar action, that is, the *pull* action, compared to the *push* action. Notably, the condition for which the stronger sensorimotor activity was detected is when the *pull* action is preceded by the *happiness* display, although this effect only emerged in post‐hoc baseline comparisons and should therefore be interpreted with some caution. Again, no effect of congruency between action and emotion was detected for the disgust expression. This finding further supports the notion that happiness displays induce distinct action predictions by 10 months of age, while disgust may be more ambiguous for young infants. This observation is consistent with the literature, where mu‐rhythm desynchronization is often associated with the familiarity of actions (Gerson et al. 2015; Vanderwert et al. 2013), dynamic emotional expressions (Quadrelli et al. 2021) and generating action predictions (Langeloh et al. 2018; Saby et al. 2012). Here, for the first time, we demonstrated that infants' sensorimotor system is sensitive both to actions and emotions: it resonates more with the action they anticipate based on an emotional context, potentially mirroring their own motor representations.

Lastly, the higher activation for the *push* action in the theta band suggest that, in a way, the action of pushing away a novel object instead of exploring it is a more unexpected outcome for infants (Köster et al. 2021) (see Supporting Information S1 results). This can be expected, given both the importance of grasping and manipulation for exploration in infancy (Babik et al. 2022) and the preference for actions directed at the body midline (Newman et al. 2001), and is in line with what was already discussed with regard to the ERPs results.

In conclusion, infants seem to interpret the further exploration of the object as the most sensible outcome, especially in the presence of positive emotionality. These results draw a first picture of the neural processing of actions embedded in an emotional context within the first year of life. They highlight the remarkable capacity of infants to combine information from multiple sources to understand the social world around them. This study also raises several questions, such as the role of other emotions and whether more complex and nuanced predictions about the association between emotions and actions emerge over time. Yet, our findings resonate with broader research on infant social cognition, which suggests that infants are remarkably expert at deciphering social cues from a very young age (Addabbo et al. 2021) and make sense of others' actions through predictive processing (Köster, Kayhan, et al. 2020). This study adds to this growing body of evidence demonstrating that infants pick up on emotional cues and use this information to make predictions about the goals and intentions behind observed actions.

### 4.1 Conclusion

The current study underscores the early emergence and complexity of social‐emotional processing in infants. By showing that infants' neural processing of others' actions is modulated by the preceding emotional information ‐ especially happiness ‐, our results converge with the idea that, early in life, human beings integrate multiple and complex social signals to navigate their social environment and develop the foundation for future social interactions.

## Author Contributions

**Elisa Roberti:** conceptualization, data curation, formal analysis, investigation, methodology, writing – original draft. **Chiara Turati:** conceptualization, methodology, supervision, writing – review and editing. **Ermanno Quadrelli:** formal analysis, methodology, visualization, writing – review and editing. **Stefanie Hoehl:** conceptualization, methodology, project administration, resources, supervision, writing – review and editing.

## Ethics Statement

The ethical committee of the University of Vienna approved the study (Approval n. 00,427, June 11, 2019), and parents gave their informed written consent before starting the experiment, according to the ethical standards of the Declaration of Helsinki (“World Medical Association Declaration of Helsinki” 2013).

## Conflicts of Interest

The authors declare no conflicts of interest.

## Supporting information

Supporting Information S1

## Data Availability

Data that support the findings of this study are openly available on the OSF platform at the following link: https://doi.org/10.17605/OSF.IO/GEFST.
