# Supplementary material for: Hold on Tight! Linking Emotions and Actions in the Infant Brain
Source: Infancy. 2025 Jul 12;30(4):e70029. doi: 10.1111/infa.70029 (PMC12255472; doi:10.1111/infa.70029)
Supplement: Supplementary file 1 — Supporting Information S1 [file INFA-30-0-s001.docx]

**Hold on tight!**  **Linking emotions and actions in the infant brain.**

**Supplementary Materials**

**Exploratory analysis in the theta band in response to the target frames.**

Theta (3-5 Hz) activation has been observed by several studies on a broader network (Köster et al., 2021; Van Der Velde et al., 2021) in response to unexpected events (Köster et al., 2019, 2021), learning processes (Begus & Bonawitz, 2020) and social processing (Van Der Velde et al., 2021). Theta can be detected around 4 Hz in infancy (3-5Hz, Reid et al., 2009; 3-6 Hz, Van Der Velde et al., 2021; 4-5 Hz, Köster et al., 2021; 4-6 Hz, Orekhova et al., 2006). The theta network tends to be wide, involving occipitoparietal and frontoparietal networks by 10 months of age (Van Der Velde et al., 2021). This rhythm is typically explored in the whole time-windows and for all regions of interest (Köster et al., 2021). For this reason, we explored theta activity in all ROIs (frontal, frontocentral, central, parietal and occipital). In particular, we expected to observe a higher theta activity in response to the more unexpected actions.

The preprocessing steps and participants included are detailed in the mu-rhythm analysis of the main document (Section 2.4 Electroencephalogram recording and analyses, time-frequency analyses paragraph).

Activation in the 3-5 Hz theta frequency band within the whole (0-1400 ms) time window was explored (**Figure S1**). An omnibus ANOVA was performed with ROI (frontal, frontocentral, central, parietal, and occipital electrodes), emotion (*happiness*, *disgust*), action (*pull*, *push*), and lateralization (left, right, and midline electrodes) as within-subject factors. A significant main effect of ROI was found, *F*(4,92)=7.75, *p*<0.001, η²_p_=0.25. The post-hoc comparison showed that, in general, the activation over the occipital region was lower (*M*= -0.58 µV; SD=0.51) than for the other regions (frontal: *M*= -0.14 µV; SD=0.49, *t*(23)=3.17, *p*=0.043, *d*=0.66, frontocentral: *M*= -0.19 µV; SD=0.44, *t*(23)=3.11, *p*=0.049, *d*=0.65, central: *M*= -0.2 µV, *t*(23)=3.44, *p*=0.011, *d*=0.72; SD=0.39, parietal: *M*= -0.15 µV; SD=0.74, *t*(23)=5.61, *p*>0.001, *d*=1.17). A main effect of action was also found, *F*(1,23)=2.69, *p*=0.025, η²_p_=0.20. The post-hoc comparison showed that for the action *push* (*M*= -0.11 µV; SD=0.39) voltage was higher than for the action *pull* (*M*= -0.40 µV; SD=0.54), indexing an overall greater theta activation for the action of pushing away the object (*t*(23)2.39, *p*=0.025, *d*= 0.49) (**Figure S2**).

The higher activation for the *push* action in the theta band suggests that pushing away a novel object instead of exploring it is a more unexpected outcome for infants (Köster et al., 2021). This can be expected, given both the importance of grasping and manipulation for exploration in infancy (Babik et al., 2022) and the preference for actions directed at the body midline (Newman et al., 2001), and is in line with what was already discussed with regard to the ERPs results.

**Figures**

**
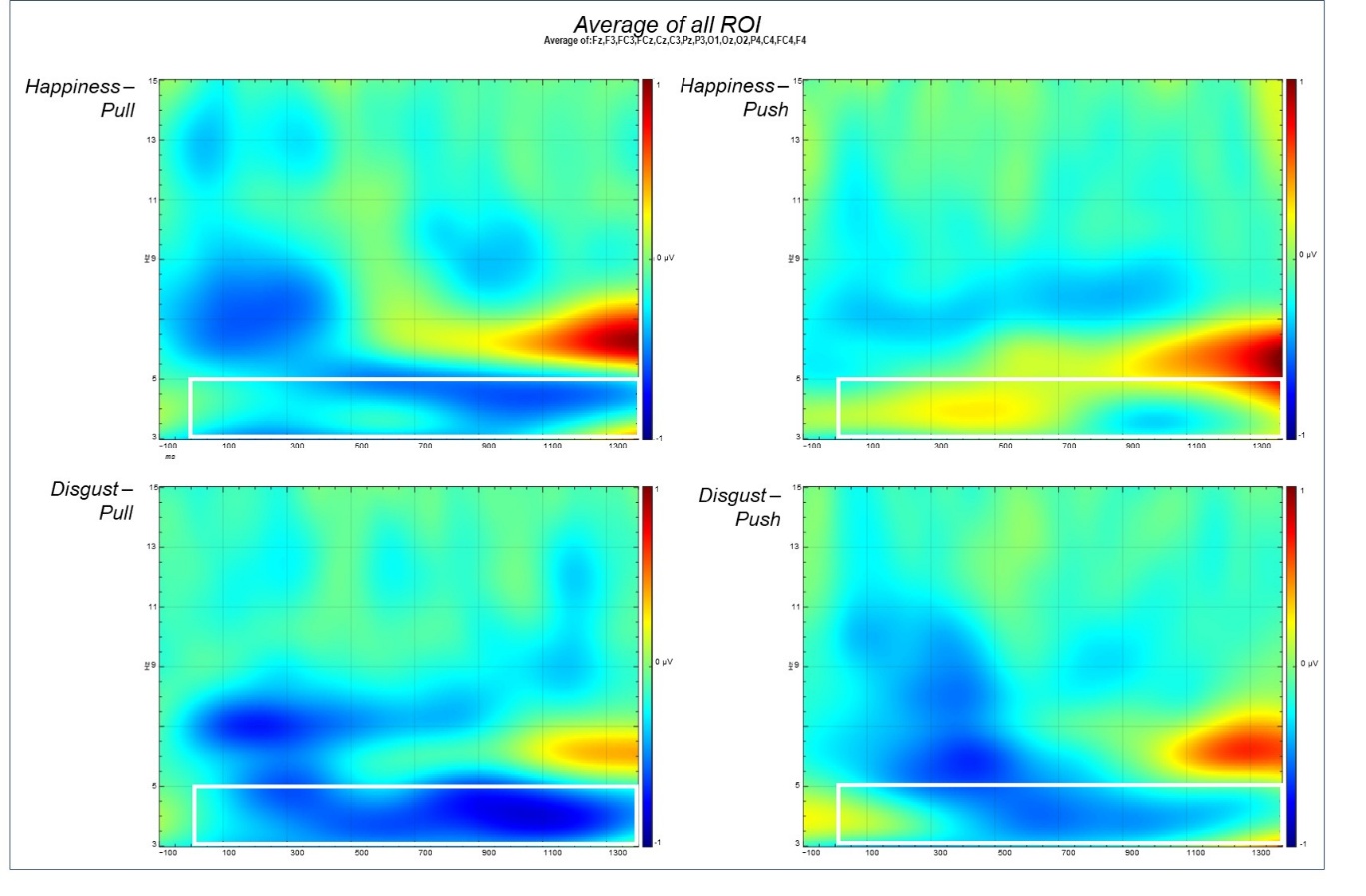
Figure S1**. Time-frequency plots for the four conditions for all ROIs. The white rectangles show the theta band analyzed (3-5 Hz) in the whole time window (0-1400 ms).

**
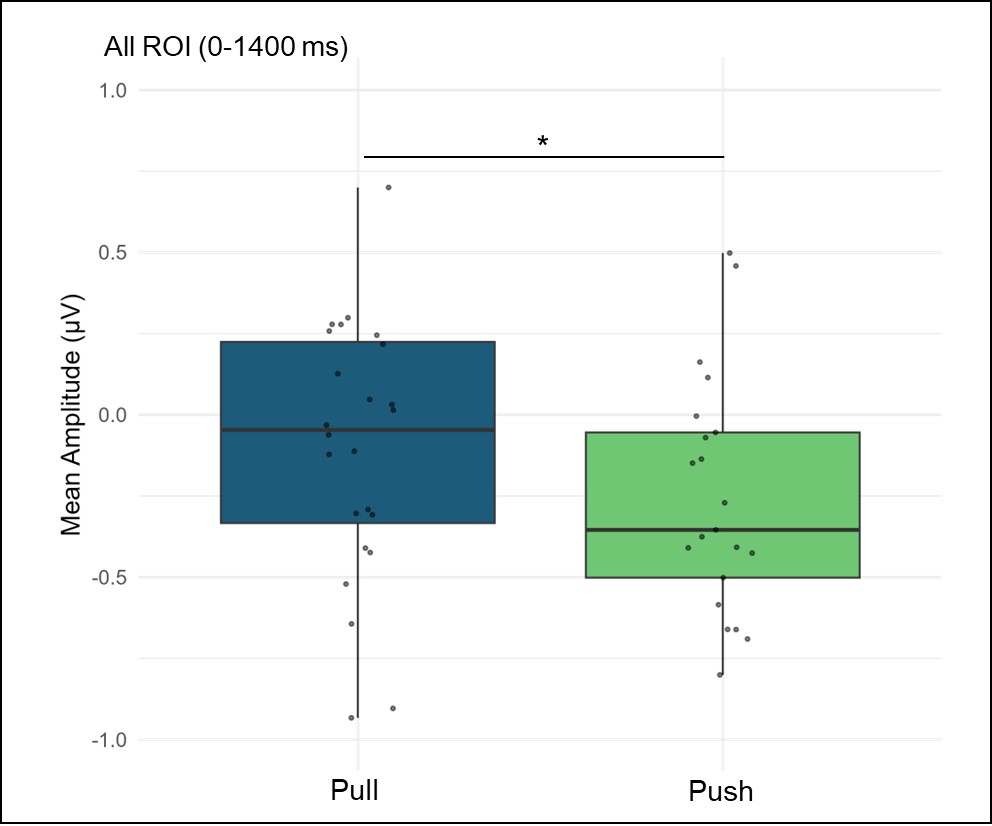
Figure S2**. Mean amplitude at all ROI for the two conditions (*pull* and *push*) in the theta band. ****p* < .001; ***p* < .01; **p* < .05.

**Bibliography**

Babik, I., Galloway, J. C., & Lobo, M. A. (2022). Early exploration of one’s own body, exploration of objects, and motor, language, and cognitive development relate dynamically across the first two years of life. *Developmental Psychology*, *58*(2), 222–235. https://doi.org/10.1037/dev0001289

Begus, K., & Bonawitz, E. (2020). The rhythm of learning: Theta oscillations as an index of active learning in infancy. *Developmental Cognitive Neuroscience*, *45*, 100810. https://doi.org/10.1016/j.dcn.2020.100810

Köster, M., Langeloh, M., & Hoehl, S. (2019). Visually Entrained Theta Oscillations Increase for Unexpected Events in the Infant Brain. *Psychological Science*, *30*(11), 1656–1663. https://doi.org/10.1177/0956797619876260

Köster, M., Langeloh, M., Michel, C., & Hoehl, S. (2021). Young infants process prediction errors at the theta rhythm. *NeuroImage*, *236*, 118074. https://doi.org/10.1016/j.neuroimage.2021.118074

Newman, C., Atkinson, J., & Braddick, O. (2001). The development of reaching and looking preferences in infants to objects of different sizes. *Developmental Psychology*, *37*(4), 561–572. https://doi.org/10.1037/0012-1649.37.4.561

Orekhova, E., Stroganova, T., Posikera, I., & Elam, M. (2006). EEG theta rhythm in infants and preschool children. *Clinical Neurophysiology*, *117*(5), 1047–1062. https://doi.org/10.1016/j.clinph.2005.12.027

Reid, V. M., Hoehl, S., Grigutsch, M., Groendahl, A., Parise, E., & Striano, T. (2009). The neural correlates of infant and adult goal prediction: Evidence for semantic processing systems. *Developmental Psychology*, *45*(3), 620–629. https://doi.org/10.1037/a0015209

Van Der Velde, B., White, T., & Kemner, C. (2021). The emergence of a theta social brain network during infancy. *NeuroImage*, *240*, 118298. https://doi.org/10.1016/j.neuroimage.2021.118298
